# Investigating the Antioxidant Potential of Mango Seed Kernel Polyphenols: Extraction and Optimization Strategies

**DOI:** 10.3390/foods15010173

**Published:** 2026-01-04

**Authors:** Poonam Choudhary, Sandeep P. Dawange, Thingujam Bidyalakshmi, Ramesh Chand Kasana, Kairam Narsaiah, Bhupendra M. Ghodki

**Affiliations:** 1 ICAR—Central Institute of Post-Harvest Engineering and Technology, Ludhiana 141004, India; dawange.sandeep@icar.gov.in (S.P.D.); bidyalakshmi.devi@icar.gov.in (T.B.); ramesh.kasana@icar.gov.in (R.C.K.); k.narsaiah@icar.gov.in (K.N.); bmg@agfe.iitkgp.ac.in (B.M.G.); 2 Division of Agricultural Engineering, Indian Council of Agricultural Research, New Delhi 110012, India; 3 Agricultural and Food Engineering Department, Indian Institute of Technology, Kharagpur 721302, India

**Keywords:** polyphenolic compounds, mango seed kernels, antioxidant and anti-bacterial activity, extraction optimization

## Abstract

Mango seed kernels, an underutilized by-product of the mango pulping industries, are a rich supplier of metabolites, specifically phenolic and flavonoid compounds. These compounds have potential health benefits. The present study aims to optimize the solvent-assisted conditions for polyphenol extraction from mango seed kernels by using the Box–Behnken design (BBD) and response surface methodology (RSM). Moreover, the effect of the solvent-to-solid ratio (5:1 to 25:1, mL/g), extraction temperature (30–70 °C), and extraction time (60–120 min) on the polyphenol yield was investigated. The optimal conditions of a solvent-to-solid ratio of 12 (mL/g), extraction temperature of 53 °C, and extraction time of 97 min showed the maximum yield of dried extract. In optimal conditions, the extract contained a total phenolic content of 110.02 ± 0.50 mg gallic acid equivalent (GAE)/g, total flavonoids of 24.58 ± 0.09 mg quercetin equivalent (QE)/g, 64.21 ± 0.12% inhibition of DPPH, and 53.25 ± 0.23% ABTS radical scavenging. Moreover, the extract at 500 mg/mL concentration showed the highest anti-bacterial activity against pathogenic bacteria of *Escherichia coli* and *Staphylococcus aureus*. Gallic acid, mangiferin, rutin, ferulic acid, cinnamic acid, and quercetin were noted in mango seed kernel extract obtained at optimal extraction conditions. Overall, a rapid and optimal methodology is reported for extracting, identifying, and quantifying polyphenols from mango seed kernels.

## 1. Introduction

The processing of fruits results in significant amounts of by-products like peels, seeds, and kernels, which are low-cost yet valuable sources of antioxidants, considered safe and potentially beneficial for therapeutic and nutritional uses [1]. Mango (*Mangifera indica* L.), a prominent tropical fruit, is consumed globally in fresh and processed form. The peel and kernel comprise 7–24% and 9–40% of the fruit’s weight, respectively, with by-products from processing accounting for 35–60% of the total weight [2]. According to an estimate, approximately 20% of the mango is processed yearly to develop value-added products such as puree, nectar, candies, jams, jellies, juice, and dried products, resulting in a bulk amount of mango peel and seed kernels. Accordingly, the waste increases constantly and generates approximately 123,000 metric tonnes annually [3]. Generally, the waste generated in the process is instantly discarded in bare ground and damp sites. The high moisture and organic matter in mango waste make it suitable for microbial growth, which causes a threat to community health and the environment. Therefore, waste disposal is an unavoidable issue for the mango processing industries. Mango seed kernels possess carbohydrates, proteins, fat/oil, and crude fiber in the range of 53.34 to 76.81%, 5.20 to 10.48%, 9.84 to 18.0%, and 0.26 to 10.60%, respectively [4]. Several studies have also reported that mango seed kernels are high in phenolic and antioxidant compounds and can be used for significant health benefits [4,5]. These bioactive molecules possess antioxidant, antimicrobial, anticarcinogenic, and anti-inflammatory properties [6]. Also, phenolic compounds have attracted significant attention due to their associated health benefits, making them highly desirable in the development of functional foods and nutraceuticals [7]. However, the optimal process for the extraction of such phenolic and antioxidant compounds remains a challenge.

Solvent-assisted extraction is the most widely used method for recovering bioactive compounds from various food matrices. This technique involves the use of solvents to extract desired compounds from solid or liquid samples. Moreover, solvent-assisted extraction is relatively simple, cost-effective, and scalable for industrial applications [7]. However, the effectiveness of the extraction is influenced by several factors, including solvent polarity, particle size, extraction temperature, time, and solvent-to-solid ratio [7]. In addition, the type of compounds that need to be recovered is critical because it influences the choice of the best extraction solvent [8,9]. The choice of solvent in solvent-assisted extraction can have an impact on the stability of bioactive compounds [9]. Some bioactive compounds are sensitive to certain solvents or extraction conditions, which can lead to chemical degradation or denaturation, thereby reducing their bioactivity or altering their properties. To overcome these challenges, optimization of extraction condition parameters is necessary. The extraction of phenolic compounds from mango peel is widely explored for use in various applications [1,2,10,11]. The extraction of bioactive compounds from mango seed kernels of a local variety of the Philippines using the solvent extraction method has been reported [7]. Unfortunately, the mango seed kernels have received very little attention in the past, especially for local varieties of Indian states. Due to growing consumer health awareness and wellness programs, the demand for natural sources of bioactive compounds has increased remarkably compared to synthetic compounds. Therefore, this study aims to explore the influence of extraction variables, viz., extraction solvent, extraction temperature, solvent-to-solid ratio, and extraction time, on the yield of phenolic compounds from mango seed kernels and extraction process optimization targeting maximum yield. The second aim was to identify and quantify the polyphenolic compounds in the mango seed kernel extract obtained under optimal conditions and to evaluate the antioxidant and anti-bacterial activity.

## 2. Materials and Methods

**Figure 1:**
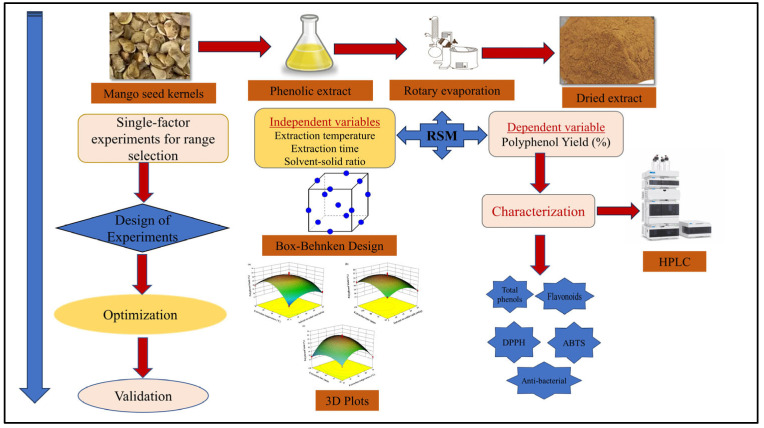
Experimental procedure.

The process for obtaining mango powder from mango seed kernels and the extraction of polyphenols is shown in Figure 1. In this section, the materials, methodology for the extraction of polyphenols, characterization of polyphenols, experimental design, and process optimization are presented in detail.

### 2.1. Ingredients and Raw Materials

Mango stones were collected from the processing and pulping industries of the Punjab province of India. The stones were carefully cleaned to remove any pulp residue before drying in the tray dryer for three days at 50 °C. The hand tool made by ICAR-Central Institute of Post-Harvest Engineering and Technology, Ludhiana, India, was used to decorticate stones to harvest kernels. Subsequently, the kernels were dried in a hot air oven for 2 days at 50 °C (Memmert UN55, Heilbronn, Germany). Dried kernels were milled by dry milling in a hammer mill. Homogeneous and uniform samples were obtained by passing the milled powder through a sieve set to obtain a 1 mm particle size. The powder samples were passed through sieve no. 16 BSS (1.19 mm). The powder samples were stored in air-tight containers in the refrigerator at 4 °C. All of the phenolic standards, HPLC-grade water, HPLC-grade acetic acid, and HPLC-grade acetonitrile, were purchased from Himedia, Mumbai, India, and MP Biomedicals, Navi Mumbai, India.

### 2.2. Extract Preparation

The dried powder of mango seed kernel (MSKs) was placed in a temperature-controlled steam-jacketed container of 200 L capacity. The samples were shaken using a shaker at 100 rpm at desired concentrations of aqueous ethanol in the temperature-controlled container. First, the extraction was performed at a specified time, temperature, and solvent-to-solid ratio according to the experimental design (presented in Section 2.3.2). In the second step, the extract was centrifuged using a decanter at 3000 rpm at 25 °C for 15 min. Finally, the supernatant was collected and kept at −80 °C until analyses were conducted. Each extraction experiment was performed in triplicate.

### 2.3. Statistical Design

Initially, experiments were conducted using a single factor or one factor at a time for screening the significant factors/parameters. The data were presented as the mean (±standard error) of three replications. The analysis of variance (ANOVA) for single-factor experiments was carried out by OPSTAT software 6.8 (CCSHAU, Hisar, India) online version [12]. Duncan’s multiple range test (*p* ≤ 0.05) was used to determine the least significant variances between the mean values of the investigated parameters.

Design Expert Software (Version 13.0) was used for experimental design, statistical analysis, process optimization, and model fitting when multiple parameters and interactions between them were considered. The Box–Behnken design (BBD) and response surface methodology (RSM) were used as statistical tools for process optimization in the software. The analysis of variance (ANOVA) method was used to examine the experimental data, and their corresponding *p*-values estimated the significance of the regression coefficients.

#### 2.3.1. Single-Factor Design

The single-factor experiments were carried out in the laboratory to examine the influence of individual parameters on total phenol content (fresh extract used) in triplicate. The impact of all four factors, ethanol concentration, extraction temperature, extraction time, and solvent-to-solid ratio, was assessed using different levels of each factor.

#### 2.3.2. Experimental Design

Based on the findings of single-factor experiments, the range of individual parameters was specified. Further, a Box–Behnken experimental design was used to optimize the extract yield from mango seed kernel extracts and ascertain the combined and interactive effect of these variables on the extraction yield. The solvent concentration was fixed at 50% ethanol throughout the experimental runs. In the present study, 17 experimental runs were carried out as per the experimental design. The three significant factors selected for this study were described as a solvent-to-solid ratio (A), extraction temperature (B), and extraction time (C) and coded into three levels, +1, 0, and −1 for high, intermediate, and low values, respectively, as shown in Table 1. The polyphenol yield (%) was taken as the response parameter. The second-order response surface model was developed by using the response and input variables in Equation (1).

(1) $$Y=\beta_{0}+\sum \beta_{i}X_{i}+\sum \beta_{ii}X_{i}^{2}+\sum_{i=1}^{n}\beta_{ij}X_{i}X_{j}+\varepsilon$$

where *Y* is the response; *β*_0_ is a constant; *β_i_* is the linear term effect; *β_ii_* is the quadratic term effect; *β_ij_* is the interaction effect; *X_i_* and *X_j_* are the coded independent factors; and *ε* is experimental error. In order to evaluate the significance of the estimated model, an analysis of variance (ANOVA) was carried out on the responses and input variables, and the effects of the linear, quadratic, and interaction terms were calculated for total polyphenol yield. The optimum conditions for input variable levels were ascertained using the desirability feature of the Design Expert Software.

### 2.4. Determination of Extract Yield

The obtained extracts were evaporated to dryness in a rotary evaporator with a water bath (Hahn shin, Gimpo-si, Republic of Korea) at 45 °C under vacuum. After complete drying, the dried extract was weighed, and extract yield was described as the amount of dried extract (g) obtained per 100 g of dried mango seed kernel powder.

### 2.5. Model Fitting and Suitability of the Model

The predictive model was obtained by fitting the second-order polynomial models, and the response value was evaluated for suitability and fitness by analysis of variance (ANOVA). The coefficient of regression (R^2^), adjusted R^2^, and lack of fit were used as good predictive model parameters to confirm the ANOVA results.

### 2.6. Validation of Optimal Conditions and Predictive Models

The effectiveness of the developed model equations for predicting the optimum response values was confirmed by performing the experiments at optimal conditions. The experiments were conducted in triplicate, and the mean data were compared with the predicted data.

### 2.7. Total Phenol Content (TPC)

The total phenolic content was determined in the dried extract using Folin–Ciocalteu’s phenol reagent [13]. The amount of total phenols was calculated from the standard curve prepared simultaneously by taking gallic acid as the standard phenol in the concentration range of 5–100 µg/mL. The findings were presented as mg gallic acid equivalents (GAE)/g dry weight.

### 2.8. Total Flavonoids Content (TFC)

TFC was measured by an aluminum chloride colorimetric assay in the dried extract [14]. Quercetin (20–100 µg/mL) was used as a standard, and the findings were reported as quercetin equivalents (mg QE)/g dry weight.

### 2.9. Antioxidant Activity

#### 2.9.1. 1,1-Diphenyl-2-picrylhydrazyl (DPPH) Assay

The antioxidant activity of the extract obtained under optimal conditions was carried out using a stable 1,1-diphenyl-2-picrylhydrazyl (DPPH) radical [15]. The DPPH dye (0.2 mM) was dissolved in 100 mL ethanol with vigorous shaking. Further, 1 mL of DPPH was mixed with 800 µL of Tris-HCl buffer (pH 7.4) and a suitable amount of extract (200 µL) in a test tube. The tubes were kept in the dark for 30 min. Then, absorbance was taken at 517 nm against a blank comprising 1.2 mL of ethanol and 800 µL of Tris-HCl buffer (pH 7.4) using a spectrophotometer (UV 1800, Shimadzu, Kyoto, Japan). The control sample was represented by 1 mL DPPH, 800 µL of Tris-HCl buffer (pH 7.4), and 200 µL of ethanol. The antioxidant activity was calculated using Equation (2).

(2) $$Scavenging\;capacity\;of\;DPPH\;(\%)=\frac{\left(A_{0}-A_{1}\right)}{A_{0}}\times 100$$

where A_0_ and A_1_ indicate the absorbance of the control and sample, respectively.

#### 2.9.2. 2,2′-Azino-bis (3-ethylbenzothiazoline-6-sulfonic acid) (ABTS) Radical Scavenging Assay

The ABTS radical scavenging activity was determined following the procedure defined by Re et al. [16]. The ABTS solution was prepared by mixing 7.0 mM ABTS and 2.45 mM potassium persulfate solution in a 1:1 ratio and then kept in the dark for 16 h. After, it was diluted with methanol to achieve an absorbance value of 0.700 at 734 nm in a spectrophotometer (UV 1800, Shimadzu, Japan) to obtain the working solution. From this working solution, 3.950 mL ABTS solution was mixed with 50 µL of extract in a test tube, kept for 30 min, and the absorbance was measured at 734 nm.

### 2.10. Anti-Bacterial Activity

The anti-bacterial activity of the MSK extract obtained under optimal conditions against pathogenic bacterial species such as *Staphylococcus aureus* MTCC 439 and *Escherichia coli* MTCC 96 was evaluated by calculating the diameter of inhibition zones. The microbes were procured from the Institute of Microbial Technology, Chandigarh, and were analyzed by agar well diffusion method [17]. The bacterial cultures were grown overnight in a nutrient broth medium at 37 °C. With a micropipette, 100 µL of each culture was spread on the surface of nutrient agar plates. Then, a 6 mm diameter well was made aseptically using a sterilized cork borer, and a 50 µL of extract solution at various concentrations (50 mg/mL to 500 mg/mL) was introduced into the well. Distilled autoclaved water served as control. After 48 h of incubation at 37 °C, the diameter of the zone of inhibition was determined in centimeters. The extracted polyphenols diffuse in the agar media and inhibit bacterial growth.

### 2.11. Chromatographic Profile of Phenolics by RP-HPLC

The mango seed kernel extract obtained under optimal conditions was subjected to RP-HPLC analysis to identify and quantify major phenolic compounds using a liquid chromatograph (Agilent 1260 Infinity, Santa Clara, CA, USA). It was equipped with a pumping system (1260 Quat Pump VL, Santa Clara, CA, USA), autosampler (1260 ALS, Santa Clara, CA, USA), reversed-phase ZORBAX Eclipse Plus C18 column (100 mm × 4.6 mm, 3.5 µm, Agilent, Santa Clara, CA, USA), and diode array detector (1260 DAD VL, Santa Clara, CA, USA). The mobile phase comprised 3% acetic acid in HPLC-grade water (A) and a mixture of acetic acid, acetonitrile, and water in the ratio of 3:50:47 (B). Both mobile phases were degassed with a vacuum pump before use in HPLC. The flow rate of the mobile phase was fixed to 1 mL/min, and the elutes were identified at a 280 nm wavelength. The gradient elution was programmed to change in four stages: (i) 0 min 100% A, (ii) 20 min 65% A, (iii) 40 min 55% A, and (iv) 50 min 30% A. All of the standard and sample solutions were filtered through a 0.45 μm polyvinylidene fluoride (PVDF) membrane filter (SFPV25X, AXIVA, Sonipat, India), and 20 μL of filtered solution was injected for chromatographic separation. Prior to injecting the next sample, the column was equilibrated with the mobile phase for 15 min. The standard gallic acid, mangiferin, ferulic acid, rutin, cinnamic acid, and quercetin were used (1 mg/mL). The identification of phenolic compounds was carried out by their specific retention time of each analyte and compared with the standard compounds run under similar conditions. In contrast, the individual phenolic compound of the sample was quantified by calculating the area under each peak of the individual compound compared with the area under the standard.

## 3. Result and Discussion

The results related to solvent-assisted extraction of polyphenols from mango seed kernels (a by-product of industries) are presented and discussed in detail in this section.

### 3.1. Selection of Significant Independent Experimental Parameters

#### 3.1.1. Influence of Ethanol Concentration

**Figure 2:**
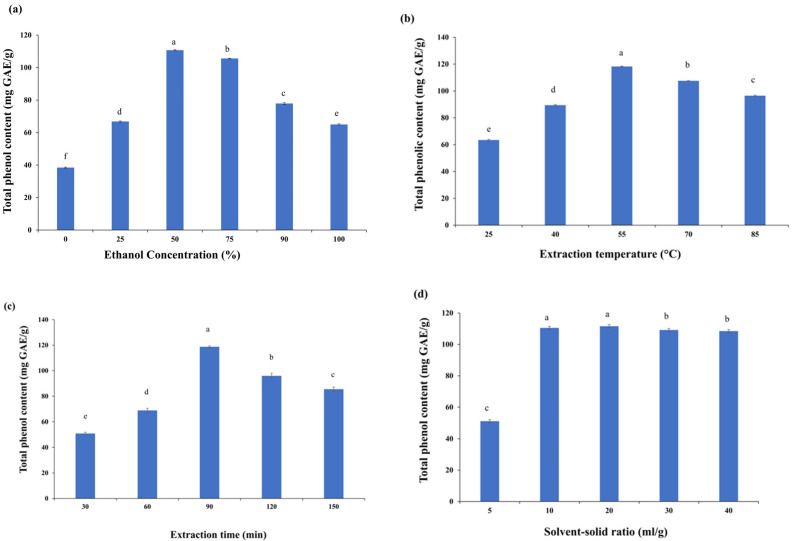
Effect of process variables on total phenolic content: (**a**) influence of ethanol concentration (%), (**b**) influence of extraction temperature (°C), (**c**) influence of extraction time (min), and (**d**) influence of solvent-to-solid ratio (mL/g). Means with same lowercase letters are not significantly different (*p* ≤ 0.05).

The impact of ethanol concentration on phenolic content varies depending on the specific source or material being studied. Different plant materials, fruits, and other biological sources possess a variety of phenolic compounds, each having their unique characteristics and solubility in ethanol. This highlights the need for process optimization for maximizing the phenolic content based on the specific material being used. In the present study, as the concentration of ethanol was increased, the amount of recovered total phenolic content in the MSK extract followed a nearly parabolic curve from 0% to 100%, as shown in Figure 2a. As the ratio of ethanol concentration in the water–ethanol binary combination increased up to 50%, the yield of total phenol increased as well. After that point, however, the yield decreased, most likely as a result of a reduction in the efficiency of ethanol at higher concentrations. In a similar study, Lim et al. [7] observed the highest value of polyphenol at a 50% ethanol concentration, whereas the lowest value was observed at a 100% concentration. Therefore, the 50% ethanol concentration was to be fixed in the polyphenol optimization extraction process using RSM. These results are consistent with the report of Kumar et al. [18]. In general, water is used for swelling plant material/samples, which assists in disrupting the bonds between the target components and substrate when mixed with a solvent like ethanol [19]. In addition, the cell membranes are readily permeable to water and low ethanol concentrations. Still, high ethanol concentrations can induce protein denaturation, which also prevents the dissolution of phenolics and reduces mass transfer [20]. Similarly, the binary mixture of ethanol and water was found to be superior to their pure solvents [21,22].

#### 3.1.2. Influence of Extraction Temperature

The optimal temperature facilitates the maximum recovery of polyphenols as they are temperature-sensitive. An increase in extraction temperature improves the extraction of phenolics by enhancing their solubility by accelerating the rates of mass transfer and diffusion [23]. However, the extraction efficiency is also adversely affected at higher extraction temperatures [24]. In the present study, when the extraction temperature was raised from 25 to 55 °C, the recovery of total phenols increased, peaked at 55 °C, and subsequently markedly decreased. At 55 °C, the recovery reached its peak of 118.23 ± 0.29 mg GAE/g, and at 70 °C, it significantly decreased by 1.1-fold (Figure 2b). Accordingly, the process parameters were further optimized at a temperature of 30–70 °C.

#### 3.1.3. Influence of Extraction Time

The recovery of extracted compounds generally increases with an increase in extraction duration because phenolic compounds require a specific amount of time to dissolve in a solvent. Nevertheless, there is a possibility that the active compounds will eventually start degrading at a longer duration [19]. Thus, the impact of extraction time on the yield of total phenolic content from mango seed kernel extract was studied at levels ranging from 30 to 150 min with a fixed solvent concentration (50% ethanol), a temperature of 50 °C, and a solvent-to-solid ratio of 10 (mL/g). Total phenol recovery increased from 30 to 100 min of extraction time, peaked at 90 min, and then markedly dropped. After 90 min, a maximum recovery of 118.77 ± 0.68 mg GAE/g was achieved, and this was followed by a marked decline of 1.23-fold (Figure 2c). Similar results have been reported in dried peach fruit powder, where the phenolic content increased up to 180 min, after which increasing extraction time did not enhance the recovery of phenolics [23].

#### 3.1.4. Influence of Solvent-to-Solid Ratio

Another factor that could be crucial for effective extractions is the solvent-to-solid ratio. With increasing solvent-to-solid ratios in the present study, a significant increase in total phenol recovery was observed, which peaked at 20 mL/g (Figure 2d). At higher levels, the recovery was almost constant. At low solvent-to-solid ratios, the phenolics in plant tissue cannot completely dissolve, resulting in lower yield in the extraction medium [25]. The phenolic content increased with increasing the solvent-to-solid ratios up to some extent [26]. As higher solvent-to-solid ratios will increase process costs, 5 to 25 mL/g was used to optimize process parameters using RSM.

### 3.2. Model Fitting and Process Optimization

The observed and predicted values of the response *Y* (extract yield, %) for all of the experimental runs, including independent parameters, are shown in Table 2. It is evident from the results that the extract yield ranged from 17.27 to 22.90 g/100 g of dried mango seed kernel powder, and the observed values showed a good correlation with the predicted values. The experimental and expected yield difference was less than 2% (Table 1). The relationship between total polyphenol yield and independent factors (*A*, *B*, and *C*) is expressed as a second-order polynomial regression equation as shown in Equation (3).

(3) $$Polyphenol\;yield=-10.37+0.66A+0.65B+0.24C-4.075E-3AB-6.25E-004AC+1.3E\;-3BC-0.015A^{2}-6.812E-3B^{2}-1.569E-3C^{2}$$

where *A*, *B*, and *C* are the coded values of the solvent-to-solid ratio, extraction temperature, and extraction time, respectively. The equation is presented in uncoded units and calculated from an average of three replicates.

### 3.3. Statistical Analysis and Suitability of the Developed Model

The outcomes of ANOVA are presented in Table 2. The model’s F-value was 405.04, and its *p*-value was less than 0.0001, which indicated that it was significant. Conversely, the F-value for lack of fit (0.9749) was more than the *p*-value, indicating that the model is non-significant to the pure error. Therefore, the developed model was found to be adequate and explained the functional relationship between the independent variables and the response values [26]. The fitness of the developed model was estimated by the regression coefficients (R^2^), adjusted R^2^ (adj. R^2^), and predicted R^2^ (pred. R^2^). The determination value of the quadratic regression model coefficient (R^2^) was 98.94%, indicating that the developed model did not explain only about 1% of the total variations. A strong correlation was reported between the actual and predicted values, which was supported by a high adjusted R^2^ (adj.) value. The value of Pred R^2^ indicates how well the model predicts a response value. The Pred. R^2^ of 0.983 is in reasonable agreement with the Adj R^2^ of 0.987. The linear, quadratic, and interactive coefficients of model terms were significant and demonstrated the presence of the interaction between the independent parameters under study (Table 3).

The suitability of the developed model was evaluated using diagnostic plots. The normal probability plots of residuals followed a straight line (Supplementary Figure S1a). The present study showed an inverted S-curve with a long tail, and except for two outliers, all points were distributed symmetrically within ±2SD. The plots of residuals against fitted values and run orders are displayed in Supplementary Figures S1b and S1c, respectively. The data points in these plots fell randomly on either side of 0, with no discernible patterns. These findings suggest that the residuals have a constant variance, are randomly distributed, and are independent. The data points on the plot of predicted values versus observed values in Supplementary Figure S1d are close to a straight line, indicating that the data from actual experiments and the data derived from the developed mathematical model are consistent. Thus, based on the outcomes of model diagnostic plots, the developed model was suitable to determine the effects of process variables for the extraction of phenolic compounds from mango seed kernels.

### 3.4. Influence of Process Variables

**Figure 3:**
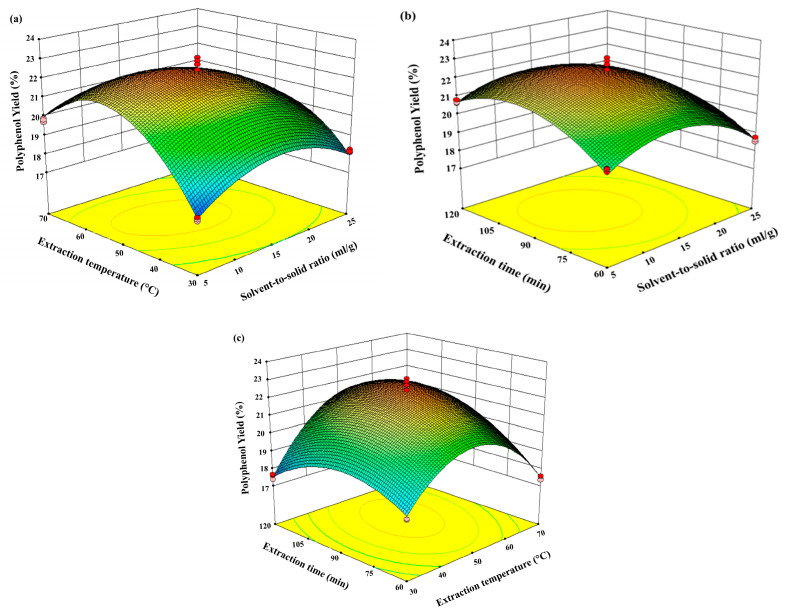
Response surface 3D plots (**a**–**c**) showing the effect of solvent-to-solid ratio (mL/g), soaking temperature (°C), and time (min) on the extraction of phenolic compounds from mango seed kernels.

According to the developed model, the three-dimensional surface plots investigated the interactive effects of the process variables on the polyphenol yield from mango seed kernels. The plot depicts the interactive effects of the solid–liquid ratio, time, and temperature on the yield of total polyphenols after drying (Figure 3). Further, increasing the solvent–solid ratios increased the yield of polyphenols up to 15 mL/g, as shown in Figure 3a,b. However, a further increase in the solvent–solid ratios did not lead to a rise in the yield of phenolic compounds. Similar to this, the yield of polyphenolic compounds increased and reached a maximum at 60 °C when the soaking temperature rose from 30 °C to 60 °C. After that, the higher temperature degraded the phenolic compounds (Figure 3a,c). The total polyphenol yield in the dried extract increased with the rise in extraction time from 60 to 120 min (Figure 3b,c).

### 3.5. Process Optimization and Model Validation

The optimal polyphenol extraction process conditions and validation data are presented in Table 4. The optimal process conditions are a temperature of 52.97 °C, a solvent–solid ratio of 12.54 mL/g, and a time of 96.55 min for polyphenol extraction. The predicted result obtained by RSM for the yield of polyphenols from mango seed kernels was 22.57%, while the validated result was 22.68 ± 0.11%. The experimental and predicted values showed a non-significant difference (*p* > 0.05) that confirmed the excellent predictive capacity of the mathematical model. Moreover, the model suitably explains the process parameters for optimizing the extraction process of phenolics from mango seed kernels. The variation in the responses is discussed further below. However, the optimum conditions as reported by Anta et al. [27] were 60 min of extraction time, a temperature of 68.7 °C, and an agitation speed of 424 rpm for extraction of polyphenol from the mango seed kernel. The difference in the optimum conditions with our study might be due to different factors, such as the solvent-to-solid ratio and solvent concentration, taken for the study.

#### 3.5.1. TPC, Flavonoids, and Antioxidant Activity at Optimized Dried Conditions

The TPC, flavonoids, and antioxidant activities are presented for dried extract obtained under optimized extraction conditions. The data recorded on the TPC and flavonoids were 110.02 ± 0.50 mg GAE/g and 24.58 ± 0.09 mg QE/g, respectively. In addition, the dried extract exhibited 64.21 ± 0.12% and 53.25 ± 0.23% of DPPH and ABTS radical scavenging activities, respectively. The TPC and flavonoid compounds and antioxidant activity depend on the genetic makeup of mango varieties and their geographical locations, maturity stages, extraction method, and post-harvest storage and processing [5,28]. TPC has been reported by various researchers and ranged from 2 to 174 mg/g in different varieties of mangoes [29,30,31].

Our results exhibited a higher yield than that of Lim et al. [7], who showed 18.19–101.68 mg/g of phenolic content. Similarly, the flavonoid content in mango seed kernels was also observed to be higher in the present study as compared to the study conducted by Saleem et al. [32], who reported a flavonoid content in the range of 2.16 to 3.11 mg CE/g. The findings of DPPH antioxidant activity in the present study are in agreement with Lim et al. [7], where the activities were reported from 3.82 to 55.61 mmol Trolox equivalent/l.

#### 3.5.2. Anti-Bacterial Activity

**Figure 4:**
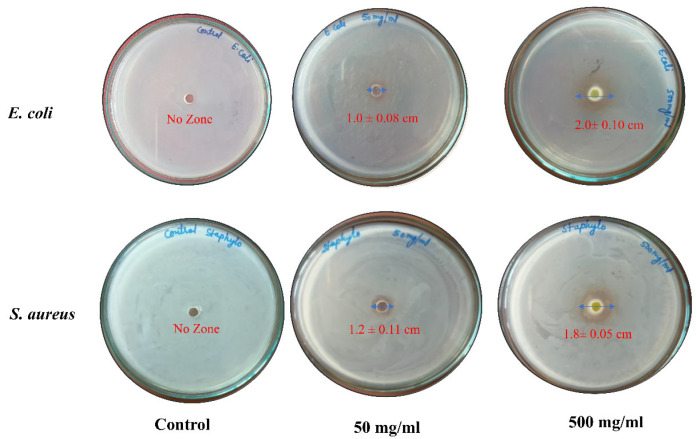
Anti-bacterial activity of mango seed kernel extract against pathogenic bacterial species Note: Numeric value in the figure shows diameter of inhibition zone as mean ± standard deviation.

The results of the anti-bacterial activity of mango seed kernel extract obtained at optimal conditions against pathogenic bacterial species are presented in Figure 4. The control samples revealed no inhibition zone, whereas the different concentrations of mango seed kernel extract exhibited dose-dependent inhibition in both the pathogenic species *E. coli* and *S. aureus*. The polyphenols present in the mango seed kernel diffuse in the agar media and inhibited the growth of the tested bacterial strains. Furthermore, the inhibition zone increased from 1.0 ± 0.08 to 2.0 ± 0.10 cm and 1.2 ± 0.11 to 1.8 ± 0.05 cm, respectively, in *E. coli* and *S. aureus* with the increase in extract concentration from 50 mg/mL to 500 mg/mL. Mango seed kernel extracts possess specific phytochemicals, including flavonoids, terpenes, tannins, and coumarins, responsible for excellent antimicrobial activity [33,34]. Similar results showing high antimicrobial, anti-biofilm, and anti-fungal activities of mango seed kernel extract have also been reported in various studies [35,36,37].

#### 3.5.3. Chromatographic Profile of Phenolics by RP-HPLC

The major phenolic compounds along with their concentrations in mango seed kernel extract obtained under optimal conditions are shown in Table 5. The quantitative analysis exhibited that the mango seed kernel possesses gallic acid, mangiferin, rutin, ferulic, cinnamic acid, and quercetin in concentrations of 137.80, 33.46, 453.95, 103.74, 14.87, and 4.21 mg/100 g, respectively. Rutin was the predominant phenolic compound found in the extract, followed by gallic acid and ferulic acid (Supplementary Figure S2). The presence of high rutin, gallic acid, and ferulic acid indicates good antioxidant properties, such as anti-inflammatory, anti-Alzheimer, anti-ulcer, and anti-cancer potential, which can protect the functions of the heart, liver, brain, etc. [38,39,40].

## 4. Conclusions

The present investigation shows that 50% (*v*/*v*) ethanol is an excellent solvent for the extraction of the highest polyphenol content from mango seed kernels compared to pure counterparts. The combined interaction provided the maximum polyphenol extract yield (22.68%) at a solvent–solid ratio of 12 mL/g, with a temperature of 53 °C for 97 min. In these optimal conditions, the extract showed a total phenolic and flavonoid content of 110.02 mg GAE/g and 24.58 mg CE/g, respectively. Nevertheless, the extract obtained under optimal conditions exhibited excellent antioxidant activity with DPPH and ABTS assays and anti-bacterial activities against pathogenic species. The RP-HPLC results of mango seed kernel extract obtained under optimal conditions revealed the presence of six specific polyphenols in adequate amounts. The concentration of phenolic compounds was reported in the order of rutin > gallic acid > ferulic acid > mangiferin > cinnamic acid > quercetin. These findings suggest that mango seed kernel extract obtained under optimal conditions possesses high antioxidant and anti-bacterial activity and could be utilized as a natural source of phenolic compounds in food, cosmetics, and various pharmaceutical applications.

## Figures and Tables

**Table 1:** Independent variables with experimental level.

Factors	Low (−1)	Middle (0)	High (+1)
Solvent-to-solid ratio (mL/g)	5	15	25
Extraction temperature (°C)	30	50	70
Extraction time (min)	60	90	120

Note: Numeric values −1, 0, and +1 indicate the low, mid, and high experimental level, in coded form, respectively.

**Table 2:** Experimental design and responses on polyphenol yield using Box–Behnken design.

Standard Order ^a^	Run Order ^b^	*A*	*B*	*C*	Polyphenol Yield (%)		Residual Error	Error, %
					Observed	Predicted		
11	1	0 (15)	−1 (30)	+1 (120)	17.55 ± 0.15	17.45	0.10	0.56
1	2	−1 (5)	−1 (30)	0 (90)	17.45 ± 0.11	17.43	0.02	0.10
14	3	0 (15)	0 (50)	0 (90)	22.36 ± 0.09	22.47	−0.11	0.47
6	4	+1 (25)	0 (50)	−1 (60)	18.63 ± 0.13	18.85	−0.22	1.14
12	5	0 (15)	+1 (70)	+1 (120)	20.13 ± 0.11	20.00	0.13	0.66
3	6	−1 (5)	+1 (70)	0 (90)	19.82 ± 0.10	20.05	−0.23	1.15
13	7	0 (15)	0 (50)	0 (90)	22.39 ± 0.07	22.47	−0.08	0.34
7	8	−1 (5)	0 (50)	+1 (120)	20.72 ± 0.10	20.64	0.08	0.40
15	9	0 (15)	0 (50)	0 (90)	22.25 ± 0.06	22.47	−0.22	0.96
9	10	0 (15)	−1 (30)	−1 (60)	17.98 ± 0.03	18.22	−0.24	1.31
8	11	+1 (25)	0 (50)	+1 (120)	18.94 ± 0.05	19.26	−0.32	1.68
10	12	0 (15)	+1 (70)	−1 (60)	17.44 ± 0.11	17.65	−0.21	1.17
17	13	0 (15)	0 (50)	0 (90)	22.90 ± 0.18	22.47	0.43	1.93
4	14	+1 (25)	+1 (70)	0 (90)	17.27 ± 0.10	17.42	−0.15	0.86
5	15	−1 (5)	0 (50)	0 (90)	19.66 ± 0.11	19.47	0.19	0.97
16	16	0 (15)	0 (50)	0 (90)	22.16 ± 0.07	22.47	−0.31	1.36
2	17	+1 (25)	−1 (30)	0 (90)	18.16 ± 0.09	18.06	0.10	0.54
Std. Dev.						0.23		
Mean						19.75		
CV (%)						1.17		
R^2^						0.989		
Adj R^2^						0.987		
Pred R^2^						0.983		

Notes: (1) The terminologies in superscripted forms represent, as follows, ^a^ randomized and ^b^ non-randomized experimental runs. (2) *A*, *B*, and *C* represent the solvent-to-solid ratio in coded units (actual value in g/mL), extraction temperature in coded units (actual value in °C), and time in coded units (actual value in min), respectively. (3) Observed data for polyphenol yield are presented as mean ± standard deviation.

**Table 3:** Analysis of variance for the response surface model of the independent variables on the extraction of total phenolic compounds from the mango seed kernel.

Source	Adj SS	df	Contribution, %	Adj MS	F Value	*p*-Value
Model	196.30	9	98.93	21.81	405.04	<0.0001
Blocks	0.020	2	0.01	0.01	0.1857	<0.0001
Linear						
A	8.09	1	4.08	8.09	150.15	<0.0001
B	4.66	1	2.35	4.66	86.45	<0.0001
C	4.93	1	2.48	4.93	91.60	<0.0001
Two-Way Interaction						
AB	7.97	1	4.02	7.97	148.02	<0.0001
AC	0.42	1	0.21	0.42	7.83	0.0079
BC	7.29	1	3.67	7.29	135.29	<0.0001
Quadratic						
A^2^	28.74	1	14.48	28.74	533.80	<0.0001
B^2^	93.81	1	47.28	93.81	1742.10	<0.0001
C^2^	25.27	1	12.74	25.27	469.24	<0.0001
Residual	2.10	39	1.06	0.054		
Lack of Fit	1.00	27	0.50	0.037	0.41	0.9749
Pure Error	1.10	12	0.55	0.092		
Cor Total	198.42	50	100			

df—degree of freedom, Adj SS—adjusted sum of squares, Adj MS—adjusted mean square.

**Table 4:** Optimal conditions for maximum extraction of polyphenol yield from mango seed kernels.

	Solvent–Solid Ratio (mL/g)	Time (min)	Temperature (°C)	Polyphenol Yield (%)
Predicted values	12.54	96.55	52.97	22.57
Observed values	12	97	53	22.68 ± 0.11

Note: Observed data for polyphenol yield are presented as mean ± standard deviation.

**Table 5:** HPLC analysis of mango seed kernel extract obtained at optimal extraction conditions.

Compounds	RT (min)	Concentration (mg/100 g)
Gallic acid	1.71	137.80 ± 0.12
Mangiferin	10.66	33.46 ± 0.21
Rutin	12.77	453.95 ± 0.08
Ferulic acid	17.75	103.74 ± 0.05
Cinnamic acid	22.91	14.84 ± 0.09
Quercetin	26.27	4.21 ± 0.01

Note: RT indicates retention time of each peak observed in RP-HPLC.

## Data Availability

The original contributions presented in the study are included in the article/Supplementary Material, further inquiries can be directed to the corresponding author.

## Supplementary Materials

The following supporting information can be downloaded at: https://www.mdpi.com/article/10.3390/foods15010173/s1, Figure S1: Model suitability diagnostic plots: (a) normal plot of residuals, (b) plot of residuals against fitted values, (c) plot of residuals against run order, and (d) plot of predicted values against observed values; Figure S2: HPLC chromatogram of mango seed kernel extract. 1: Gallic acid; 2: mangiferin; 3: rutin; 4: ferulic acid; 5: cinnamic acid; 6: quercetin.
