# Supplementary material for: Investigating the Antioxidant Potential of Mango Seed Kernel Polyphenols: Extraction and Optimization Strategies
Source: Foods. 2026 Jan 4;15(1):173. doi: 10.3390/foods15010173 (PMC12786019; doi:10.3390/foods15010173)
Supplement: Supplementary file 1 [file foods-15-00173-s001.zip › foods-3162422-supplementary.pdf]

Supplementary Information

(a)

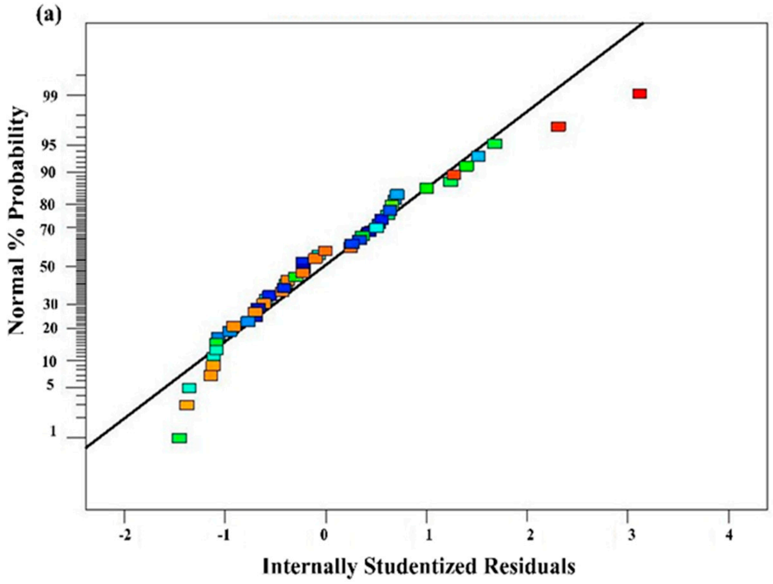

(b)

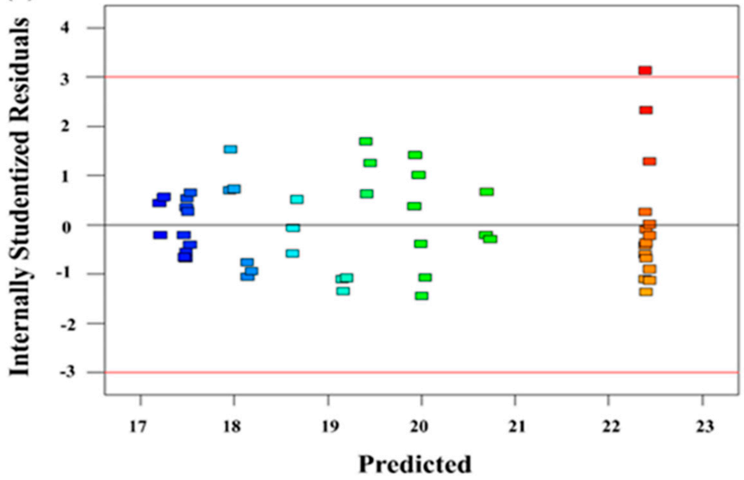

(c)

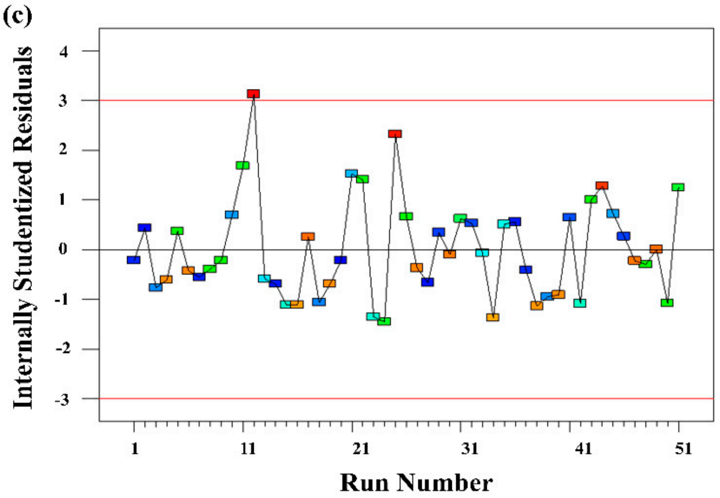

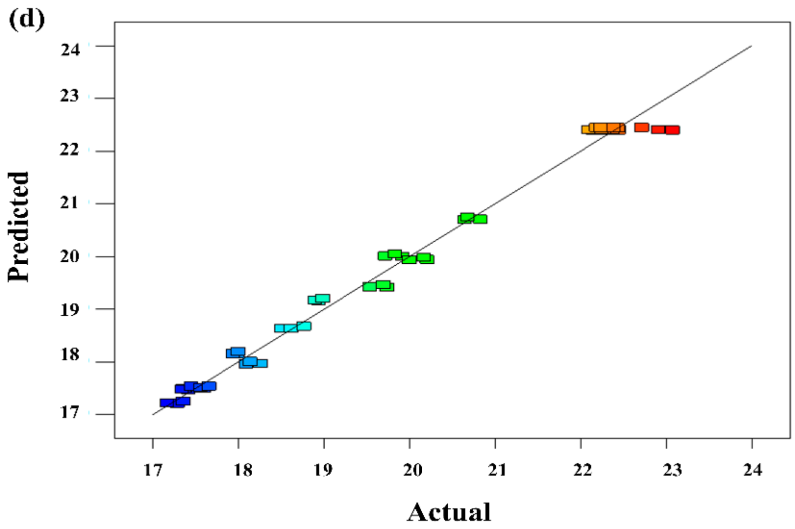

Figure S1. Model suitability diagnostic plots: (a) normal plot of residuals, (b) plot of residuals against fitted values, (c) plot of residuals against run order, and (d) plot of predicted values against observed values.

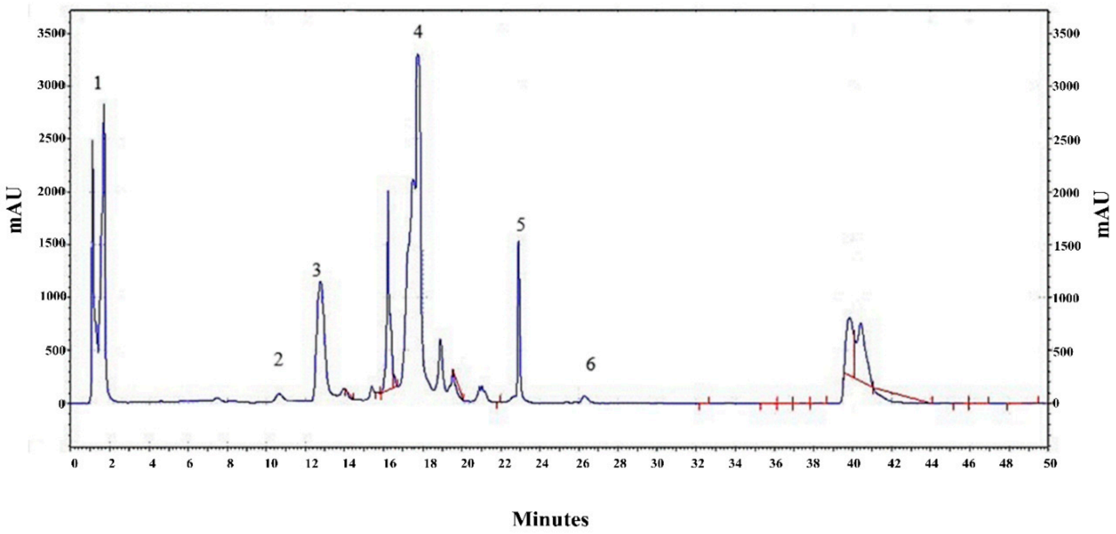

Figure S2. HPLC chromatogram of mango seed kernel extract. 1: Gallic acid; 2: Mangiferin; 3: Rutin; 4: Ferulic acid; 5: Cinnamic acid; 6: Quercetin.
